# Corpus Callosum Agenesis: An Insight into the Etiology and Spectrum of Symptoms

**DOI:** 10.3390/brainsci10090625

**Published:** 2020-09-09

**Authors:** Jagoda Hofman, Michał Hutny, Karolina Sztuba, Justyna Paprocka

**Affiliations:** 1 Students’ Scientific Society, Department of Pediatric Neurology, Faculty of Medical Science in Katowice, Medical University of Silesia, 40-752 Katowice, Poland; jagoda.hofman98@gmail.com (J.H.); michal.j.hutny@gmail.com (M.H.); k.sztuba96@gmail.com (K.S.); 2 Department of Pediatric Neurology, Faculty of Medical Science in Katowice, Medical University of Silesia, 40-752 Katowice, Poland

**Keywords:** corpus callosum agenesis, children, genetic background, clinical symptoms

## Abstract

Brain hemispheres are connected by commissural structures, which consist of white matter fiber tracts that spread excitatory stimuli to various regions of the cortex. This allows an interaction between the two cerebral halves. The largest commissure is the corpus callosum (CC) which is located inferior to the longitudinal fissure, serving as its lower border. Sometimes this structure is not completely developed, which results in the condition known as agenesis of the corpus callosum (ACC). The aim of this paper was to review the latest discoveries related to the genetic and metabolic background of ACC, including the genotype/phenotype correlations as well as the clinical and imaging symptomatology. Due to various factors, including genetic defects and metabolic diseases, the development of CC may be impaired in many ways, which results in complete or partial ACC. This creates several clinical implications, depending on the specificity of the malformation and other defects in patients. Epilepsy, motor impairment and intellectual disability are the most prevalent. However, an asymptomatic course of the disease is even more common. ACC presents with characteristic images on ultrasound and magnetic resonance imaging (MRI).

## 1. Introduction

**Figure 1:**
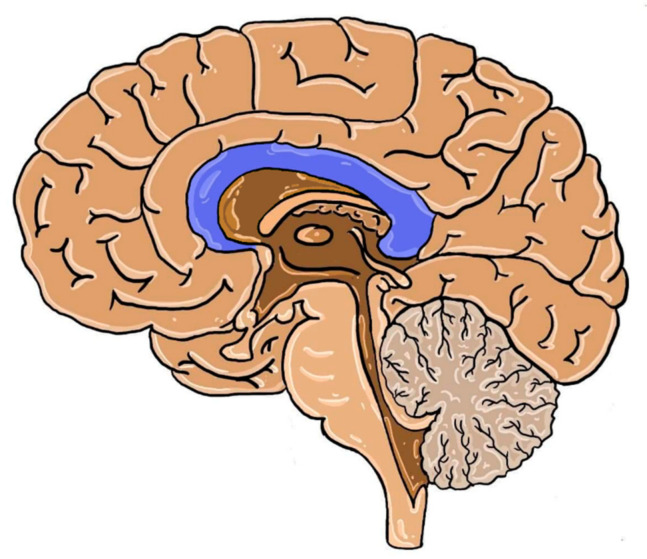
The structures of the human brain in the median plane. Dark blue: corpus callosum.

Cerebral hemispheres are connected by neuronal fibers organized in larger tracts i.e., anterior and posterior commissures, hippocampal commissure and the fornix. The largest of them is the corpus callosum (CC). It is the largest white matter structure of the human brain, which is located at the bottom of the longitudinal fissure, also serving as the cover for the lateral ventricles. It consists of axons that pass signals to various regions of the contralateral hemisphere cortex [1]. Even though the number of neurons is steady at birth, their further development (myelination and redirection) continues in the postnatal period. Thickness of CC, fiber cross-section and bundle size increases through childhood and adolescence with slight differences among sexes [2,3]. The localization of CC in the human brain is presented below in Figure 1.

Fetal development of this structure may be interrupted by various genetic factors and maternal alcohol abuse [4]. The most frequent causes of corpus callosum agenesis (ACC) are gene mutations that are related to pathways of axon guidance, ciliary development, cell adhesion, proliferation, differentiation and migration. The presence of Probst bundles is common evidence of abnormal commissure formation. These bundles are packs of longitudinally (rather than transversely) oriented neurons. As a result, they are unable to fulfill their role of connecting both hemispheres. Imaging techniques and morphometrics also provide evidence of multiple levels of callosal developmental anomalies from thinning to complete agenesis. The absence of all parts of CC is identified as complete ACC, as opposed to the absence of some parts (partial ACC). This malformation may be an isolated abnormality, but it may be also a component of syndromes composed of various neurological pathologies [4,5].

The prevalence of ACC in the general population varies depending on the sources and is probably underestimated often due to the asymptomatic course. The usual range is 1:5000 to 1:4000 (0.020–0.025%) [6] although higher prevalence (0.2–0.7%) is also reported [7,8]. In subjects with impaired neurodevelopment, this defect is present in even up to 1–3% of a given group [8,9]. The genetic cause is identifiable for 30–45% of ACC cases, with approximately 10% of them having chromosomal abnormalities and the remaining 20–35% being single gene mutations [10]. Even though the identification of complete ACC does not raise much doubt, the exact definitions of partial ACC are matters of debate, thus creating the difficulty in comparing the occurrence of the two. The dispute frequently involves the inclusion of hypoplastic CC into the pool of partial ACC. The summary of several studies considering the percentages of complete and partial ACC is given in Table 1 [3,6,8,9,11].

The following paper presents the summary of the results of the latest research on ACC. The collected data were divided into sections, representing various aspects related to the disorder, i.e., etiology, spectrum of symptoms and neuroimaging.

## 2. Material and Methods

The search strategy consisted of controlled vocabulary and keywords. The following databases were searched: PubMed, PubMed Central and Medline. The main search concept was to combine “corpus callosum agenesis” with the related terms, such as “etiology”, “genetic etiology”, “ciliopathies”, “treatment”, “imaging” and “metabolic disorder”. Filters which were applied to limit the retrieval included the language and the date of publication in particular. For this reason, only English language papers published within the previous 5 years were considered for this review. The Online Mendelian Inheritance in Man (OMIM) database was used as the reference for gene and mutation symbols, chronology of research, location and phenotype identification.

## 3. Results

### 3.1. Etiology

Agenesis of the corpus callosum (ACC) has a varied background, ranging from maternal drinking, which is responsible for many defects in neural development [4], to prenatal infections, chromosomal errors, or gene mutations [12]. The latter can present as isolated ACC or can be combined with other malformations that lead to syndromes (e.g., Coffin–Siris Syndrome) [13]. In the following part, we present the latest findings related to genes and their mutations responsible for the occurrence of ACC based on clinical studies and animal models, using genetic techniques such as whole-exome sequencing (WES).

#### 3.1.1. Genes Linked to Isolated ACC

##### Recessive *CDK5RAP2* Variants (9q33.2; MIM 604804)

Cyclin-dependent kinase 5 regulatory subunit 2 (*CDK5RAP2*) is a pericentriolar structural component that organizes microtubules in centrosomes. By the Hippo pathway, it regulates proliferation, differentiation and migration in developing organs, mainly influencing the quantity of cells, rather than the pattern of the tissue. Tissue growth is limited by phosphorylation of Yes-associated protein (YAP) and transcriptional co-activator with PDZ-binding motif (TAZ) [10,14,15]. *CDK5RAP2* mutations are linked to primary microcephaly. However, these mutations might be also responsible for ACC [10,14,15]. In 2016, three siblings (each with radiologically diagnosed isolated ACC) were examined using the WES technique, which led to the discovery of compound heterozygous variants in *CDK5RAP2* that were not present in their unaffected parents [10].

##### DCC Netrin-1 Receptors in the Development of Commissural Axons

The *DCC* gene (18q21.2; MIM 120470) encodes transmembrane proteins in commissural axons, which enhances cell adhesion [16]. They are engaged in connecting hemispheres by commissures by binding netrin-1 (NTN-1) that functions both locally and diffusely in the developing brain. The phenotype differs depending on the type of mutation in the genotype of the organism. However, each presentation is seen as a version of the novel syndrome i.e., Mirror Movements 1 (MRMV1; MIM 157600) [16,17]. Monoallelic frameshift, nonsense and (in some cases) missense mutations of the *DCC* gene result in decreased binding of NTN-1 to DCC. Monoallelic frameshift and nonsense mutations are more frequently associated with a congenital phenotype of mirror movements compared to missense mutations of *DCC*, which are rather linked to the isolated phenotype of ACC. In the latter case, DCC proteins may be non-functional and hypomorphic. Monoallelic frameshift and missense mutations may also present with a mixed phenotype, i.e., mirror movements with isolated ACC [16,17].

#### 3.1.2. Genes Recently Studied on Murine Models Concerning Their Role in Neurodevelopment

##### Ephrin Receptor Genes (*EphB1*; 3q22.2; MIM 600600)(*EphB2*; 1p36.12; MIM 600997)

*EphB1* plays a role in regulating the migration of striatal interneurons, controlled by the Nkx2-1 factor [18]. By binding to tumor necrosis factor receptor 2, tumor necrosis factor α (TNF-α) upregulates the expression of the EphB2 receptor in neurons during neuroregeneration, which suggests a possible role of EphB2 in this process [19]. The roles of intracellular domains of EphB1 and EphB2 in the formation of CC were analyzed in 2018 in a study using ephrin receptor mutant knock-in mice. In mutant mice, they consisted of the correct surface domain, capable of supporting reverse signaling, and a β-galactosidase coding region instead of the intracellular kinase domain, thus disabling them to send the signal. The results confirmed that both types of ephrin receptors are important for the normal development of CC and anterior commissure (AC). A combination of homozygous mutations resulted in the occurrence of ACC in over 90% of animals, mostly in the caudal region. The caudal location was more specific for the EphB2 mutation rather than EphB1. It is possible that these receptors might share the genetic pathway, as the combination of heterozygous mutation of one receptor with the homozygous mutation of the other presented with a stronger phenotype of the homozygous gene [20]. Heterozygous mutations alone did not present with any ACC [19].

##### Lim Homeodomain Transcription Factor (Lhx2) in the Formation of the Glial Wedge (GW)

In 2015, a study on animal models assessed the impact of the *Lhx2* (*9q33.3* MIM 603759) gene knockout on cortical neurogenesis [21]. Although the stratification of the cortex remained intact, all layers appeared to be significantly thinner due to shorter proliferation and neurogenesis in neurons in the radial column. It was suggested that Lhx2 delayed the initiation of neurogenic differentiation [21]. Inactivation of the Lhx2 transcription factor results in a failure in GW formation, which is essential for the normal connection of hemispheres with cortical axons in rostral CC, and the absence of the hippocampal commissure whose function in caudal CC is analogous to GW’s in rostral CC, thus presenting with ACC. Previous studies also showed that Lhx2 was important for the maintenance of radial glia progenitors in the hippocampus and the neocortex [22].

##### Nogo Receptor Deficiency (NgR1; *Rtn4r*; 22q11.21; MIM 605566), (NgR2; *Rtn4rl2*; 11q12.1; MIM 610462), (NgR3; *Rtn4rl1*; 17p13.3; MIM 610461)

In 2017, creation of the NgR-null mice led to abnormal formation of the medial brain, namely the fasciola cinereum and CC with no differences in cortex size and lamination compared to wild type mice [23]. All genetically modified animals and none of the wild type subjects showed ACC with impairment of sensorimotor coordination. Nogo receptors seem to be involved in CC formation, although the precise molecular mechanism of this correlation remains unresolved [23].

#### 3.1.3. Genes Linked to Ciliopathies

##### *CDK10* gene (16q24.3; MIM 603464) Mutation Resulting in a Novel Ciliopathy Phenotype

An 11-year-old girl of Ashkenazi Jewish ancestry with a range of defects, including ACC, retinitis pigmentosa, hearing loss and short stature, was examined using the WES technique in 2018. The sequencing identified a deleterious mutation of the *CDK10* gene. The cyclin-dependent kinase 10 (*CDK10*), which is its product, is the binding partner of cyclin M (CycM), which in turn is encoded by the *FAM58A* gene (Xq28; MIM 300708) [24]. Together they form the CDK10/CycM protein kinase, which regulates ciliogenesis. Mutation of CycM is present in STAR syndrome (STAR; Xq28; MIM 300707), which shares one symptom with the presented patient, i.e., facial dysmorphism. The above symptoms are characteristic of common ciliopathies such as Bardet–Biedl syndrome (BBS1; 11q13.2; MIM 209900) or Alström syndrome (ALMS; 2p13.1; MIM 203800) [24].

##### *Kif7* (15q26.1; MIM 611254) and *C5orf42* (5p13.2; MIM 614571) Genes in Acrocallosal Syndrome (ACLS; 15q26.1; MIM 200990)

The *Kif7* gene product is crucial in the Sonic hedgehog (SHH) pathway. Its homozygous or compound heterozygous mutations, the former of which was recently identified in a six-month-old Tunisian boy with ACLS, lead to the spectrum of defects characteristic of this syndrome, such as macrocephaly, prominent forehead, depressed nasal bridge, and hypertelorism. ACLS belongs to the group of ciliopathies related to Joubert syndrome (JBTS; MIM 213300) [25,26]. Due to a broad range of phenotypes in patients with *Kif7* mutations, it may be useful to identify a group of *Kif7*-related ciliopathies. *C5orf42* (HGNC approved gene symbol: *CPLANE1*) mutations were also recently found in ACLS patients. Interestingly, a follow-up study showed persistent severe intellectual disability in *Kif7*-mutated patients, while *C5orf42*-mutated patients showed improvement from severe to mild intellectual disability. Dysfunction in these genes leads to impaired axon guidance due to ciliary function disorders related to SHH pathways [26].

##### Novel Phenotype of Joubert Syndrome (*JBTS33*; 13q21.3-q22.1; MIM 617767)—*PIBF1* (13q21.3-q22.1; MIM 607532) Insertion

Thinning of CC, though present in a two-year-old patient with Joubert Syndrome, is not typical of this disease. JBTS subjects usually demonstrate intellectual disability, hypotonia, facial dysmorphism, retinal dystrophy, cystic fibrosis, kidney disease and molar tooth sign on brain imaging, which is the result of the deepened interpeduncular fossa, thickened elongated superior cerebellar peduncles, and the absence or hypoplasia of the cerebellar vermis [27,28]. Next generation sequencing (NGS) combined with quantitative fluorescence PCR were performed in this patient. The results showed an insertion in exon 9 of *PIBF1*, which is the mutation encountered in five previous cases of JBTS. Unfortunately, further assessment of the connection between the genetical anomaly and atypicality of symptoms in this case could not be performed due to patient non-compliance [27].

#### 3.1.4. ACC Presenting in Novel Congenital Syndromes

##### 1q43q44 Microdeletion Syndrome (1q43-q44; MIM 612337)

A vast spectrum of neurological disorders may be caused by deletions in the subtelomeric region of the long arm of chromosome 1, referred to as 1q43q44 microdeletion syndrome. Patients with this disorder usually present with microcephaly, a wide range of callosal anomalies from thinning to complete agenesis, intellectual disability, and epileptic seizures. Recent studies identified three genes responsible for the development of each feature of this syndrome. *AKT3* (1q43-q44; MIM 611223) deletions lead to partially penetrant microcephaly although the contribution of *ZBTB18* (1q44; MIM 608433) and *HNRNPU* (1q44; MIM 602869) mutation is possible. In this syndrome, the presence of ACC is mostly associated with the loss of the *ZBTB18* allele, although the combination of the deletion of *ZBTB18* and *HNRNPU* genes increases the prevalence and penetrance of ACC. *HNRNPU* is also believed to be the reason behind epilepsy in the affected population [29,30].

##### Vici Syndrome (VICIS; 18q12.3-q21.1; MIM 242840)

Recently, a study on a large cohort of 50 patients assessed the correlations between the genotype and the phenotype in Vici syndrome. Due to the distribution of mutations throughout the whole coding sequence of the *EPG5* gene (18q12.3-q21.1), the precise correlation was not identified, although homozygous subjects showed a shorter lifespan (median: nine months) compared to heterozygous subjects (median: 48 months) [31]. The mutation responsible for VICIS is usually a truncating substitution mutation of a single nucleotide. ACC was present in each case. However, the direct influence of the mutation on the development of this defect was not established in that study [31].

##### Mowat–Wilson Syndrome (MOWS, 2q22.3; MIM 235730)

The *ZEB2* gene (2q22.3; MIM 605802) encodes the zinc finger E-box binding homeobox 2 (ZEB2), an important transcription factor involved in neural crest formation. Its mutations are linked to MOWS, which presents with morphological features (hypertelorism, rounded nasal tip) as well as intellectual disability, epilepsy and a wide range of brain developmental defects such as ACC, CC hypoplasia, ventriculomegaly and impaired hippocampal formation [32]. The grade of ACC was significantly different (*p* = 0.0095) between the mutation types: cACC was the most common in mutations leading to the synthesis of the defective protein, while it was rare in the cases of the absence of the protein [32].

##### Crouzon Syndrome with Acanthosis Nigricans (CAN; 4p16.3; MIM 612247)

This rare disease is caused by missense mutations in the *FGFR3* gene (4p16.3; MIM 134934) encoding fibroblast growth factor receptor 3 as opposed to the differently located *FGFR2* gene (10q26.13; MIM 176943) which is linked to the classic phenotype of Crouzon syndrome (CFD1; 10q26.13; MIM 123500). CAN shares the clinical symptoms with CFD1-craniosynostosis and facial dysmorphism (hypertelorism, upper jaw defects, exophthalmos) with skeletal abnormalities and hypopigmentation specific of CAN, which results from the atypical form of acanthosis nigricans. In 2016, the case of a 10-month-old girl presenting with the typical morphological features of CAN revealed a new heterozygous A391E type mutation (c.1172C>A) in the *FGFR3* gene inherited in autosomal dominant pattern. However, the mutation in the patient was sporadic [33]. MR imaging showed a hypoplastic CC and inferior vermis, which is a phenotype that had not been previously reported in CAN. The relationship between this finding and the pathomechanism of the main disease remains unsolved [33].

#### 3.1.5. New Discoveries in Well-Established Congenital ACC Syndromes

##### Coffin–Siris Syndrome (CSS)

This genetic disorder is caused by de novo mutations of *ARID1A* (CSS2; 1p36.11; MIM 614607), *ARID1B* (CSS1; 6q25.3; MIM 135900), *DPF2* (CSS7; 11q13.1; MIM 618027), *SMARCA4* (CSS4; 19p13.2; MIM 614609), *SMARCB1* (CSS3; 22q11.23; MIM 614608) or *SMARCE1* (CSS5; 17q21.2; MIM 616938) genes. Symptoms vary, depending on the location and the type of mutation. *SMARCB1* truncating monoallelic mutations do not lead to defects in neurogenesis, as the lack of protein product is substituted by the non-affected allele. In the case of germline missense or nonsense mutations, the result is an increased risk of schwannomatosis (exon 1, 2 or 3′ untranslated region mutations), although it was not found in Coffin–Siris syndrome subjects. In 2019, it was found that partial loss of *SMARCB1* function by monoallelic alterations was impossible to be covered with normal mRNA production from the intact allele, which leads to neurodevelopmental dysfunctions (including ACC), intellectual disability (exon 2) and choroid plexus hyperplasia (CPH) (exons 4 and 9). The latter may be considered predictive for the presence of CSS-causing mutation [13].

##### Syndromic Phenotype of ACC in Baraitser–Winter Syndrome 2 (BRWS2; 17q25.3; MIM 614583)

Gamma-actin is a cytoskeletal protein present in all cells, as all actins play a vital role in cell life through the mechanisms of cell division and movement. It is encoded by the *ACTG1* gene (17q25.3; MIM 102560) whose mutations lead to BRWS2, which is characterized by a range of neural developmental defects, including pachygyria, subcortical band heterotopia and ACC [34]. Recent post-mortem cerebral examination of a fetus with BRWS2 showed the absence of synaptosomal-associated protein (SNAP-25) positive neuronal fibers that are crucial for guidance in commissural axons. In periventricular white matter heterotopia, the GABAergic neurons were identified using interneuron markers, whereas the glutamatergic neurons were identified with anti-SNAP-25 staining. Such alterations were not present in the age-matched control, thus providing evidence for the involvement of the *ACTG1* gene mutation in the pathomechanism of BRWS2 [34].

##### Andermann Syndrome (ACCPN; 15q14; MIM 218000)

The characteristic features of Andermann syndrome, also known as agenesis of the corpus callosum with peripheral neuropathy (ACCPN), include agenesis of CC and early-onset polyneuropathy reflected by hypotonia and muscular atrophy. Therefore, it is a neurodevelopmental and neurodegenerative disorder. Recently a study conducted on eight subjects resulted in the identification of the genetic etiology of this disease—the expression of the *SLC12A6* gene (15q14; MIM 604878) is essential for normal prenatal axonal development, as well as the physiological function of axons in the postnatal period. The most frequent mutation in the subjects was a guanine deletion in exon 18. The product of this gene is the potassium chloride cotransporter KCC3. The neurodegenerative process in this syndrome is unusual. It does not affect the surrounding glia or neurons, nor does it show the signs of Wallerian degeneration. It is present in both peripheral and central parts of the nervous system, resulting in the loss of peripheral and cerebral neurons [35].

##### ACC and Ophthalmic Pathologies—Aicardi Syndrome (AIC; Xp22; MIM 304050)

AIC is an X-linked dominant inherited disease, mostly seen in female subjects. The condition presents with the triad of symptoms, i.e., ACC, chorioretinal lacunae and infantile spasms. Recently, it was suggested that microphthalmia should also be included [36]. Its incidence ranges from 1:110,000 to 1:93,000 live births [37,38]. It was previously thought to be caused by mutations in the *TEAD1* (11p15.3; MIM 189967) gene. However, in 2017, a study on 38 AIC subjects identified the *TEAD1* variant in only one patient, which suggested the contribution of other genes in the development of this condition [39]. Due to the lack of precise genetic or molecular possibilities for AIC identification, the diagnosis is mainly based on imaging and clinical symptoms, particularly disc anomalies (drusen, pigmented and morning glory discs) [38]. Findings of female fetal MRI examination may be considered predictive [37].

#### 3.1.6. Genes Linked to Metabolic-Related ACC

##### *EARS2* (16p12.2; MIM 612799) Linked with the Combined Oxidative Phosphorylation Deficiency 12 (COXPD12; MIM 614924)

Mitochondrial glutamyl-tRNA synthetase is an enzyme crucial for the translation of various mitochondrial mRNAs to proteins. It is encoded by the *EARS2* gene, thus the missense mutation variants of this gene lead to the impairment of mitochondrial function in the affected tissues, which in turn might develop into mitochondrial disorders, i.e., leukoencephalopathy or leukodystrophy. The increase in lactate is a direct consequence of this defect. Twenty-six patients were diagnosed with recently discovered lethal neonatal pathology (COXPD12) presenting with a characteristic imaging pattern (including callosal hypogenesis in each case) and clinical features of various severity. Studies failed to identify the exact genotype–phenotype correlation between mutation types or anomalies in brain morphology and the severity of the disorder [40,41].

*EARS2* mutations were also found in vitro in a patient with leukoencephalopathy, brain calcifications and cysts (LCC; MIM 614924), which is a disease different from COXPD12 in terms of neuroimaging and clinical symptoms. The genetic background of LCC is also different from COXPD12; the former is caused by mutations in the *SNORD118* gene (17p13.1; MIM 614561) rather than in *EARS2*. Even though the symptoms and imaging results (including CC atrophy) were typical of a patient with LCC, the fibroblasts of the examined child showed mitochondrial dysfunction due to a decreased *EARS2* protein level. The level of lactate was also increased, which is a feature characteristic of COXPD12. Interestingly, according to the American College of Medical Genetics and Genomics (ACMG), the *EARS2* variant found in this subject is considered pathogenic and should result in the phenotype typical of COXPD12. It demonstrates that the results of in vitro sequencing do not necessarily reflect the expected phenotype in vivo, as the possible influence of genetic or epigenetic factors in some cases remains unknown [42].

##### Defects in Mitochondrial NADP Metabolism Causing CNS Abnormalities

The *NADK2* gene (5p13.2; MIM 615787) is responsible for the functioning of mitochondrial NAD+ kinase. Its mutations are linked to 2,4-dienoyl-CoA reductase deficiency (DECRD, 5p13.2; MIM 615787) due to the absence of the source of NADP [43]. Toxic influence on mitochondria is believed to be caused by the accumulation of lysin metabolites (e.g., saccharopine) rather than lysine [44]. A recent study presented a patient with *NADK2* mutation in whom the thinning of CC and the delay in myelination were found on imaging examination for the first time in the case of DECRD. Metabolic features of the subject initially indicated familial hyperlysinemia (hyperlysinemia, type I; 7q31.32; MIM 238700), which is caused by a mutation in the *AASS* gene (7q31.32; MIM 605113), which subsequently was ruled out in order to consider other possible etiologies [43]. On the other hand, one study suggested that the wide phenotype range of NADK2 deficiency due to *NADK2* mutations might be the result of not only hyperlysinemia or DECRD, but could be also influenced by other NADPH-dependent processes [45].

##### Pyridoxine-Dependent Epilepsy (EPD; 5q23.2; MIM 266100)

Mutations in the *ALDH7A1* gene (5q23.2; MIM 107323) encoding α-amino-adipic semialdehyde (α-AASA) dehydrogenase (antiquitin) lead to the disorder of lysine metabolism, which in turn causes epilepsy that poorly responds to therapy with anticonvulsants. The spectrum of mutations differs among populations. Recently, a novel mutation (c.393+1G>A) was identified in a Tunisian boy [46]. One of the characteristic symptoms is the presence of lysine metabolites in blood. Although seizures can be controlled with pyridoxine, the metabolite levels do not change throughout therapy [47]. Antiquitin is also present in the radial glia and plays a role in the early development of the CNS, which is also associated with structural brain lesions in EPD patients, the most common being hypoplasia, partial agenesis, or dysplasia of CC (34% of patients in the study population of 44 subjects) [48].

In the future, the above findings might serve as the targets of prenatal screening tests or possibly gene therapy. Early detection of neural developmental anomalies allows for the planning of relevant therapies for predicted symptoms in advance or discovering more severe defects underlying the malformation. Adequate dietary modifications in patients with metabolic diseases may prevent the full development of the disease [49,50].

All previously mentioned genes and syndromes connected with ACC are given in Table 2.

### 3.2. Spectrum of Symptoms

#### 3.2.1. Clinical Features

Agenesis of CC, which is the largest white matter tract in the human brain, is one of the most common cerebral malformations. Patients affected by this abnormality present with a varying range of symptoms, some being severely impaired, while others may not even be aware of the abnormality since it does not interfere with their normal functioning. The prevalence of ACC may be higher because mostly symptomatic cases are reported. That being so, a non-symptomatic course with or without incomplete CC may be of common occurrence.

##### Factors Defining Severity

As already mentioned, the neurodevelopmental outcome may considerably vary between individuals affected with ACC [51]. The severity of the symptoms differs depending on whether it is partial (pACC) or complete (cACC) agenesis of CC and whether it is an isolated case of ACC or it is rather linked to other abnormalities [9,51]. It should be noted that age also plays an important role in the clinical picture of the patient. The end of childhood and coming into adolescence can be the time when the symptoms start to show, even if the child previously showed normal intelligence and neuropsychological development, particularly in the case of isolated ACC. During that period, the development of CC and its myelination are completed, making the compensating potential of other cerebral commissures insufficient. That is the reason why each individual affected with asymptomatic ACC needs a strict annual follow-up [52].

##### Intellectual Development

The corpus callosum connects the hemispheres, which allows higher cognitive processes and emotional as well as social functioning in humans. These abilities are impaired in patients with autism. ACC and autism share some common symptoms, such as difficulties in abstract reasoning, communication and social skills, affective prosody, and emotional and behavioral problems. It encouraged the researchers to explore the connection between the changes in CC and autism spectrum disorders [53]. Most studies did show the occurrence of agenesis or the reduction/thinning of CC in the cases of autism. One of the studies showed that the average thickness of CC was 15% less in autistic subjects than in the healthy control group [51,54].

Although according to the meta-analysis of 27 studies, 76.04% of children prenatally diagnosed with isolated cACC had normal neurodevelopment, developmental delay and retardation in achieving milestones are commonly reported by parents of children with ACC [51]. Extracallosal abnormalities increase the risk of other problems, such as speech delay or epilepsy.

##### Motor Function and Homeostasis

The muscle tone in patients with ACC can be normal, increased (hypertonia) or decreased (hypotonia). In the case of the thinning of CC, axial hypotonia was detected [55]. Additionally, appendicular hypertonia can occur. The same inconsistency applies to the size of the head. Patients are reported to present with microcephaly or macrocephaly. However, normocephaly is also found in the ACC population [9]. Fetuses with an isolated case of agenesis are at a higher risk of chromosomal anomalies [51], including trisomies, deletions, missense, nonsense and truncating mutations and frameshifts [56,57].

Agenesis of CC can cause alterations in the central osmoregulatory system. Although ACC that results in hyponatremia is an extremely rare connection, such cases have been recently reported (i.e., the first report of an adult with hyponatremia due to the syndrome of inappropriate antidiuretic hormone hypersecretion (SIAD)). A 41-year-old woman with a very mild clinical picture of ACC presented with chronic hyponatremia, plasma hypo-osmolality and urinary hyper-osmolality with an increased level of plasmatic antidiuretic hormone (ADH). At the same time, the patient was diagnosed with Sjögren syndrome, systemic lupus erythematosus (SLE) and systemic arterial hypertension. Bone density measurements indicated osteoporosis, even though the patient had not undergone menopause yet. Wide diagnostic procedures were performed. However, no connection was found between the patient’s diseases and hyponatremia. It was assumed that in this rare case of the concomitance of ACC and SIAD, the above diseases were the aggravating factors, which caused chronic hyponatremia. It can result in neurological symptoms such as cognitive deficit, impairment of mobility and attention deficit [57].

### 3.3. Neuroimaging

**Figure 2:**
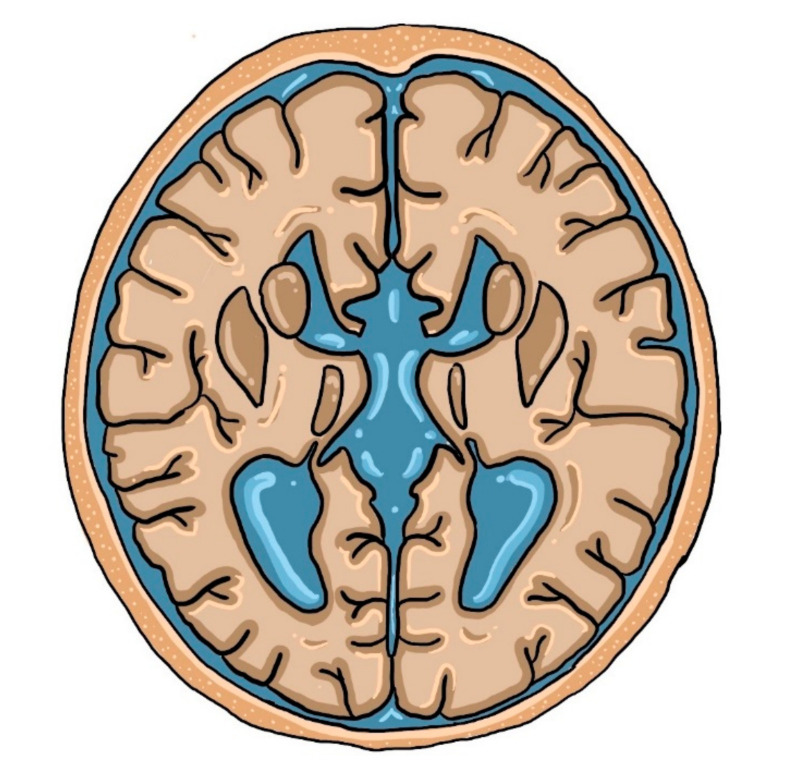
The ‘racing car sign’ created by enlarged ventricles.

Although fetal imaging studies are usually conducted in the transverse plane, the midsagittal view is more preferable for ACC screening. The follow-up studies proved that up to 80% of prenatally identified isolated ACC cases develop normally. Tractography with a special MRI technique (Diffusion-Weighted MRI; DWI) is a more reliable clinical predictive tool, which provides the graphic representation of white matter corticospinal tracts in the brain [49,50]. Colpocephaly is a common finding in neuroimaging studies [9]. However, this is not the most characteristic neuroimaging finding on MRI in patients with ACC. A ‘racing car sign’, formed by ventriculomegaly and the absence of CC with intervening Probst bundles, is a typical imaging symptom of this developmental defect, as presented below in Figure 2.

The Probst bundles, also known as longitudinal callosal fascicles, are white matter fibers that physiologically cross CC. When CC is absent, neuronal fibers develop parallel to the interhemispheric fissure rather than perpendicular to it. Along with dilated lateral ventricles, they create an image of a racing car that was mentioned above. The higher the percentage of the Probst bundles, the better the adaptive and social functions are [9,58].

**Figure 3:**
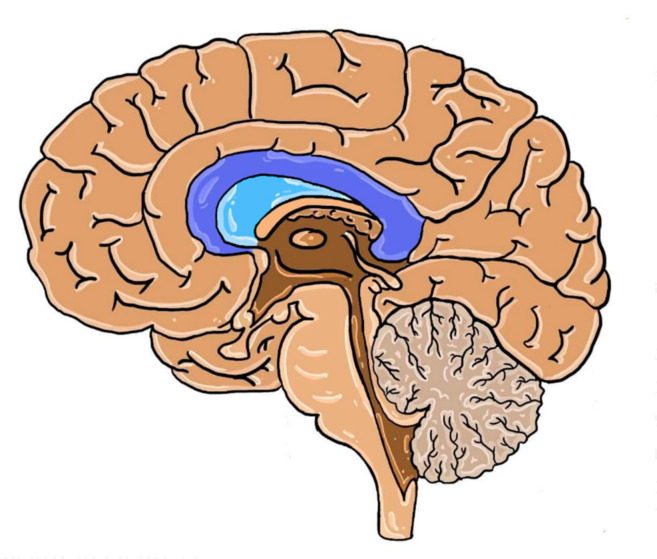
Human brain structures in the medial plane with the septum pellucidum (in color). Dark blue: corpus callosum; light blue: septum pellucidum.

The septum pellucidum (SP) is a structure located in the midline of the brain, which serves as the medial wall of the lateral ventricles in the vicinity of CC, as seen in Figure 3.

Even though an abnormal septum pellucidum (ASP) is not typical of each case of pACC, it can be a potential indicator of this developmental defect due to the frequent presence of ventriculomegaly [59]. When obtaining high-quality images of the brain in the midsagittal plane poses some difficulty, as in the case of fetal screening, the indirect signs as ASP may prove to be useful. This sign was present in 52% of isolated pACC cases in the study population. The prevalence of ASP was also different and depended on the range of callosal defect (see Table 3) [60,61].

Other parameters useful in the diagnosis of ACC are the size and shape of the cavum septi pellucidi (CSP). A recent study showed the relationship between the length-to-width ratio of SP and the presence of pACC. It showed that the decrease in the length and the increase in width, as well as a non-rectangular shape, were associated with callosal dysmorphism [62]. Other structures connecting the two hemispheres are rarely completely absent in isolated ACC. In most cases, either hippocampal or anterior commissure is absent. Thus, any commissure defect seen on fetal ultrasound should be further assessed by MRI, as it bears a potential risk of ACC [61,63]. The number of patients with additional defects in the study are presented in Table 4 [61].

Starting from the second trimester of pregnancy, distinctive differences can be found in isolated ACC cases as seen on MRIs of brain sulci. Those alterations are different from the patterns present in the developing fetal cortex. Furthermore, they are independent of cerebral growth and do not return to their physiological shape [64].

## 4. Conclusions

With the rapid advances in molecular biology techniques of mutation detection and identification, research on the genetic etiology of brain malformations has also become available. In the case of ACC, the impairment usually occurs in pathways connected with axonal growth, cell migration and neuron connection. Various possible grip points result in a highly diverse clinical picture of ACC patients in both CC morphology (complete, partial) and symptoms (isolated, in syndromes). Another genetic factor contributing to heterogeneity of callosal defects is the variety of possible mutation types, depending on its result (loss of function, impaired formation). Unfortunately, not all examinations resulted in precise genotype–phenotype correlations.

Moreover, mutations may affect CC not only by congenital development malformations, but they can also lead to metabolic diseases (mostly linked to mitochondrial metabolism), resulting in delayed myelination and degeneration. Although the morphological defects are permanent, the clinical features such as seizures are possible to overcome with proper diet control.

Most cases are asymptomatic, and the symptoms usually become apparent at the beginning of adolescence. Different types of ACC present with slightly distinct clinical conditions, although the general range of symptoms consists of abnormal fine motor control, epilepsy (including poorly-responsive EPD) and abnormal cognitive status (in some syndromes, there is an identified link with autism or intellectual disability). The characteristic abnormalities are found on imaging, i.e., Probst bundles are a sign of impaired commissural axon guidance and growth, representing the lack of connection between hemispheres. Callosal dysmorphisms usually present with developmental defects of other interhemispheric connections, i.e., anterior or hippocampal commissures. Assessment of these anatomical structures, SP and the shape and dimensions of its cavity provide useful information indicating ACC, even if it is not detected in the imaging studies.

To sum up, ACC is a malformation with varied clinical presentation, depending on multiple factors. Its prenatal detection using imaging techniques and genetic analysis enables future planning of adequate care, which is particularly crucial in cases of metabolic diseases.

## Figures and Tables

**Table 1:** Comparison of the occurrence of complete and partial corpus callosum agenesis (ACC) in five studies.

No.	Country	Number of Subjects (of Which cACC or pACC)	cACC (%)	pACC (%)	*p*-Value ^a^	Imaging Technique
1. [6]	France	25 (22)	68	20	<0.005	MRI
2. [3]	Israel	50 (32)	40 ^b^	24 ^b^	>0.050	-
3. [8]	Iran	18 (5)	11.1	16.7	>0.050	MRI
4. [9]	Canada	125 (105)	52	32 ^c^	<0.010	MRI
5. [11]	France	34 (34)	76	24	<0.005	US + MRI

Note: ^a^ *p*-value for the one-sample test of proportion (null hypothesis: H_0_: *p* = 0.5; alternative hypothesis: H_1_: *p* ≠ 0.5); ^b^ results of post-mortem examination; ^c^ in the population of pACC, a significant difference between the occurrence of anterior ACC (only completely developed splenium; 2%) and the posterior ACC was found (missing or hypoplastic splenium fibers; 30%). Abbreviations: cACC, complete agenesis of the corpus callosum; pACC, partial agenesis of the corpus callosum; US, ultrasonography; MRI, magnetic resonance imaging.

**Table 2:** Comparison of recently discovered genes and syndromes linked to ACC and previously known syndromes resulting in ACC.

Genes and Syndromes Recently Linked to ACC			
Name	First Detection (Years)		Connection with ACC (Years)
*CDK5RAP2*	1993 ^a^		2016 [10]
*DCC* (MRMV1)	1988 ^a^ (2010) ^a^		2017 [16] (2017) [16]
*EphB1*	1995 ^a^		2018 [20]
*EphB2*	1991 ^a^		2018 [20]
*Lhx2*	1993 ^a^		2015 [22]
*Rtn4r*	2001 ^a^		2017 [23]
*Rtn4rl2*	2003 ^a^		2017 [23]
*Rtn4rl1*	2003 ^a^		2017 [23]
*CDK10*	1994 ^a^		2018 [24]
*Kif7* (ACLS)	2004 ^a^ (1980) ^a^		2011 ^a^
*C5orf42* (ACLS)	2012 ^a^ (1980) ^a^		2018 [26]
*PIBF1* (JBTS33)	2002 ^a^ (2015) [27]		2015 [27] (2015) [27]
(1q43q44 microdeletion syndrome)	(1985) ^a^		(2001) ^a^
*AKT3*	1999 ^a^		2007 ^a^
*ZBTB18*	1997 ^a^		2014 ^a^
*HNRNPU*	1992 ^a^		2017 [30]
*EPG5* (VICIS)	2000 ^a^ (1999) ^a^		2013 ^a^ (1999) ^a^
*FGFR3* (CAN)	1991 ^a^ (1992) ^a^		2008 ^a^ (2016) [33]
*SMARCB1* (CSS3)	1998 ^a^ (2012) ^a^		2013 ^a^ (2013) ^a^
*EARS2* (COXPD12)	1999 ^a^ (2012) ^a^		2013 ^a^ (2013) ^a^
*NADK2* (DECRD)	2012 ^a^ (1990) ^a^		2016 [43] (2016) [43]
Syndromes previously associated with ACC			
Name	First discovery	First ACC connection found	Recent discovery
*ZEB2* (MOWS)	1998 ^a^ (2002) ^a^	2001 ^a^ (2002) ^a^	2017 (2017) [32]
*ACTG1* (BRWS2)	1987 ^a^ (1998) ^a^	2012 ^a^ (2012) ^a^	2019 (2019) [34]
*SLC12A6* (ACCPN)	1999 ^a^ (1972) ^a^	2002 ^a^ (1977) ^a^	2016 (2016) [35]
*TEAD1* (AIC)	1991 ^a^ (1969) ^a^	NS (1994) ^a^	2017 (2017) [39]
*ALDH7A1* (EPD)	1994 ^a^ (1954) ^a^	NS (NS)	2017 (2017) [46]

Note: ^a^ Data collected using the OMIM database. Information can be found using the MIM code for each gene/phenotype placed above in appropriate subsections of the paper. Abbreviations: ACC, agenesis of the corpus callosum; ACCPN, ACLS, acrocallosal syndrome; Andermann syndrome; AIC, Aicardi syndrome; BRWS2, Baraitser–Winter syndrome 2; CAN, Crouzon syndrome with acanthosis nigricans; COXPD12, combined oxidative phosphorylation deficiency 12; CSS3, Coffin–Siris syndrome; DECRD, 2,4-dienoyl-CoA reductase deficiency; EPD, pyridoxine-dependent epilepsy; JBTS33, Joubert syndrome; MOWS, Mowat–Wilson syndrome; MRM 1, mirror movements 1; NS, Not specified; VICIS, Vici syndrome.

**Table 3:** Percentage of patients with normally shaped SP in the study population (depending on the type of pACC) [60,61].

Type of Defect	Percentage of Patients with Normal SP (%)
**Abnormal Splenium**	70
**Abnormal Rostrum**	72
**Short CC**	69
**Multiple Abnormal Segments**	32

Abbreviations: CC, corpus callosum; pACC, partial agenesis of the corpus callosum; SP, septum pellucidum.

**Table 4:** Summary of commissural defects in the patient population [60].

Type of Defect	Number of Patients	Percentage (%)
No forebrain commissure	3	4.8
AC only	23	37.1
Residual VHC and rudimentary CC	16	25.8

Abbreviations: AC, anterior commissure; VHC, vestigial hippocampal commissure; CC, corpus callosum.
